# Walking Human Detection Using Stereo Camera Based on Feature Classification Algorithm of Second Re-projection Error

**DOI:** 10.3389/fnbot.2019.00105

**Published:** 2019-12-18

**Authors:** Shuhuan Wen, Sen Wang, ZhiShang Zhang, Xuebo Zhang, Dan Zhang

**Affiliations:** ^1^Key Lab of Industrial Computer Control Engineering of Hebei Province, Yanshan University, Qinhuangdao, China; ^2^Institute of Robotics and Automatic Information System, Nankai University, Tianjin, China; ^3^Department of Mechanical Engineering, York University, Toronto, ON, Canada

**Keywords:** feature target, classification, dynamic environments, SLAM, re-projection error

## Abstract

This paper presents a feature classification method based on vision sensor in dynamic environment. Aiming at the detected targets, a double-projection error based on orb and surf is proposed, which combines texture constraints and region constraints to achieve accurate feature classification in four different environments. For dynamic targets with different velocities, the proposed classification framework can effectively reduce the impact of large-area moving targets. The algorithm can classify static and dynamic feature objects and optimize the conversion relationship between frames only through visual sensors. The experimental results show that the proposed algorithm is superior to other algorithms in both static and dynamic environments.

## Introduction

Simultaneous localization and mapping (SLAM) is a basic problem and research hotspot in the field of mobile robot research (Klein and Murray, 2007; Mur-Artal et al., 2015; Mur-Artal and Tardos, 2016; Saeedi et al., 2016). It is also one of the important conditions for mobile robot to realize autonomous navigation. The existing SLAM algorithm is mainly applied to static environment (Wen et al., 2015). However, the real environment is complex and changeable, such as people walking, door switching, changes of lighting, etc. They will bring unpredictable noise for modeling and positioning of the environment, so the existing SLAM algorithm is not very suitable for dynamic environment.

There are three main problems for SLAM of mobile robots in dynamic environment: SLAM solution, data association, and dynamic target processing.

For processing dynamic targets, there are some methods with good robust performance. For example, detected dynamic points and uncertain points regarded as abnormal points are discarded (Saeedi et al., 2016). But the traditional SLAM algorithm will result in a large deviation or even failure when the moving object is too large and the moving speed is too fast or too slow.

So, this paper studied detection method of the target with different speed and different size in dynamic environment. The feature targets are classified and the relationship between frame and frame is optimized only by a visual sensor. The classification method proposed in this paper can adapt all kinds of dynamic environment, and it can process for dynamic objectives with different speed and reduce the impact of moving objects with large area. The visual sensor can be used in hand or in different platforms.

The proposed algorithm in the paper can be applied to the following field: wearable equipment, intelligent mobile terminal equipment, micro-aircraft, and intelligent robots. In the paper, a novel second re-projection error algorithm based on the texture detection is proposed. The texture detection is integrated into a target classification algorithm, which can optimize the re-projection error algorithm. The main idea of the re-projection error algorithm is that the projection point should be moved away from the corresponding feature point when dynamic feature point of the previous frame is projected onto the current frame image. For static feature points, this re-projection error should be small. In addition, the texture detection proposed in this experiment mainly adopts the idea of interframe difference. According to the different attributes of static and dynamic feature targets, the continuous frame information of the environment is scanned and captured by using the visual sensor, and the difference and weighted information is used to complete the target detection. In the paper, the two algorithms are optimized and fused with the corresponding constraints, which can realize the classification of static and dynamic feature target points and the optimization of the relationship between frame and frame. In order to verify the real-time and validness of the proposed algorithm, the ORB and SURF feature algorithms is used to extract the environmental information. The experiments are done under four different dynamic environments, including near distance targets under static background, far distance targets under static background, one dynamic target under dynamic background and multiple dynamic targets under dynamic background. The experiment results show that the proposed targets separation method of the feature points integrated with the texture detection in this experiment can successfully separate dynamic targets from static targets based on ORB and SURF feature extraction methods under four different dynamic environment. It can also complete the optimization of the relationship between frame and frame. The proposed separation method of the feature points in the paper can obtain good effect in different dynamic environments.

The remainder of the paper is organized as follows. In section Related Work, the background studied in this paper and related work are introduced. The overview of the system is presented in section System Overview. The static feature detection and pose optimization algorithm proposed in the paper are detailed in section Static Feature Detection and Pose Optimization. The realization of dynamic target detection algorithm is introduced in section Constraint. In section Experiment Results, experiments and results in different environment are given. Concluding remarks are given in section Conclusion and Future Work.

## Related Work

Moving object detection is one of the hotspots in machine vision research (Zhang et al., 2014, 2016; Choi and Maurer, 2016; Naiel et al., 2017). Fischler and Bolles (1981) proposed a paradigm for model fitting with applications to image analysis and automated cartography. According to the status of a camera, it can be divided into the static background detection and the dynamic background detection. In the static background detection, the camera is always stationary, so the moving target detection is easy. It has been widely used in scene monitoring of fixed environment, such as factory, road, and airport. The common background models have an adaptive background model based on the kernel density estimation, the Gaussian background model and the hidden Markov background model. In the dynamic background detection, the position of the camera changes, which can result in the change of the background and object in the image at the same time. So it increases the difficulty of moving target detection, which is the focus of the current research for moving target detection. There are three main categories about dynamic background detection of moving objects (Xu et al., 2011; Yin et al., 2016): optical flow, background comparison, and interframe difference method. For the optical flow method, because the background and the moving speed of the detected target is different, it can result in a large difference in optical flow. So moving objects can be identified according to it. The calculation of the optical flow is large, and there exists pore size problem. The comparison method of the background adopts image registration to dynamically update the background model, and it can compare the actual image with updated the background model to obtain the moving target. The interframe difference method is used to register the background of several successive images. The target detection is transformed into the moving object detection problem in the static background, and the moving object is separated by the difference image of the front and rear frames. The background image registration method includes texture algorithm, Fourier transform method, and feature matching method (Naiel et al., 2017). The feature matching method is simple and fast, but the matching error of the existing matching method is influenced by the changing environment.

Some previous research didn't consider the dynamic scene, and the detected dynamic or uncertain points were discarded as abnormal values (Williams et al., 2007; Paz et al., 2008; Liang and Min, 2013; Zhang et al., 2015; Zhou et al., 2015). However, when some of the dynamic objects are large, they would have large error, or even failure. In the dynamic environment, the existing research mainly adopts the filter approach (Hahnel et al., 2003) and has been successfully applied to solve the problems of SLAM based on laser scanner and radar system, but the research of applying to visual SLAM is seldom studied. Fang et al. (2019) detect and recognize the target through visual tactile fusion. Gao and Zhang (2017) explains the basics of SLAM. In Chen and Cai (2011), the visual sensor and the radar were used to detect the dynamic object. The uncertain factor is detected by using the eight-neighborhood rolling window method based on the map difference method. But the long-time static target is difficult to be detected out accurately. Zhang et al. (2018, 2019), Afiq et al. (2019) applied dynamic target detection to crowd action and emotion recognition. In addition, the lack of laser radar can bring instable judgment for some obstacles with special material (such as glass doors), which can affect the accuracy of the map (Einhorn and Gross, 2015), achieved estimation tracking of the dynamic object by combining the normal distribution transformation with the grid map. But there are some restrictions on the scope of adaptation. In Sun et al. (2017), a novel movement removal method based on RGB-D data was proposed, which enhanced the application in dynamic environment. The work of Lee and Myung (2014) and Wang et al. (2016) used the posture structure diagram and the RGB-D sensor to complete the detection of the low speed target, and re-optimized the structure of posture to obtain the corrected location and mapping results. These dynamic separation methods are still not suitable very well for the detected target with speed and volume. And the judgment is still not accurate enough or they require a high economy. In Zou and Tan (2013), the cooperation of multiple cameras is used to detect dynamic points. The separation between static feature points and uncertain points is made by a camera at first. Then multiple cameras are used to further determine the uncertainty points. The method reduces the impact of the large-scale moving objects on the system, and it is suitable for high dynamic object. The method proposed in this paper is based on Zou and Tan (2013).

In this experiment, a camera is used to solve classification problem of the feature points. Comparing with other sensors, the camera is passive, compact, and energy-saving. It has an important advantage for intelligent platforms with limited weight and energy capacity. The proposed algorithm in this experiment can achieve the detection classification of the moving objects with different speeds in a variety of complex environment, and complete the pose transformation optimization only by a visual sensor.

## System Overview

**Figure 1:**
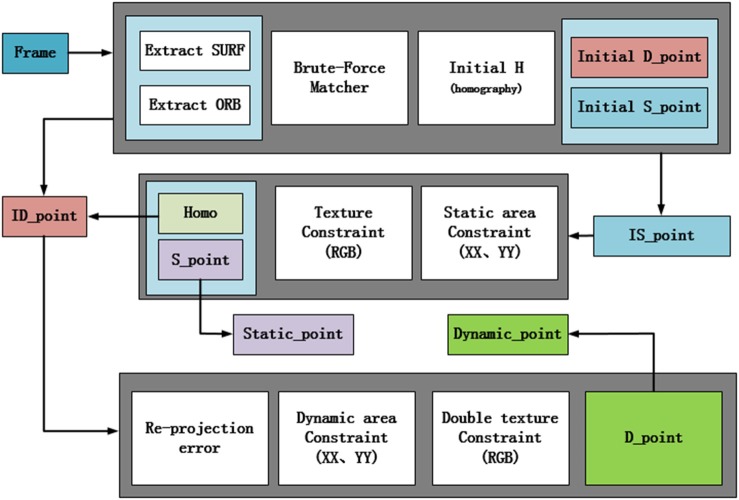
System structure.

In this experiment, a visual sensor can be used to obtain the environment information. The frame image obtained by the visual sensor is regarded as the input of the whole system framework. By calculating the transformation matrix between frame and frame, the dynamic feature points and static feature points are finally judged by the re-projection error. The framework of this system is divided into three parts shown in Figure 1. The first part is the initialized process, in which the feature of each frame image is extracted and the image relationship between frame and frame is calculated. The feature extraction of image frames has two methods. One is SURF feature extraction (Bay et al., 2006). SURF algorithm with robustness and fast speed is an enhanced version of the SIFT algorithm (Lowe, 2004). The other is ORB feature extraction algorithm (Rublee et al., 2011), which combines the FAST feature detection method with the BRIEF feature descriptor. It improves and optimizes the original two algorithms. It has a invariable scale, and the speed is faster 10 times than SURF. Different feature extraction is chosen according to the different scenes. After obtaining the feature points, the feature points can be matched by the existing Brute Force method. The matched result is passed to the homography matrix frame to obtain the transformation relation between frame and frame. By this transformation relation, the re-projection error is calculated to obtain an initialized static feature point and dynamic feature point. And the next two parts is based on the initial work.

In the paper, the dynamic and static region constraint methods are proposed for different feature attributes, and the texture detection is incorporated to realize screening data. The re-projection error method is used again when the dynamic feature point is judged, which is also the second re-projection error method proposed in this paper. It can realize the classification of dynamic target and the relationship optimization between frame and frame. The second part is filtered out static point in the second frame and the static region constraint is used to reduce false matching. The second judgment based on the texture detection is used to process the determined static point, which can achieve the separation of static points finally. After the separation of the static points, the exact static matching point is given to the homography matrix again, and then an optimal relation matrix between the frame and frame is obtained. Through this relation matrix, the uncertain points can be judged twice. The results will be used in the third part of the system. Thus, the separation of static points, the relationship optimization between frame and frame and the second judgment of uncertain points are finished, which realizes a rough judgment for the dynamic points. The third part is the separation of the dynamic feature points. The main modules are divided into dynamic region constraints and double texture detection fusion. Although the dynamic points and static points separation have the similar principle, the means of implementation are different. In the next section, the principles, methods, and parameters of each frame will be introduced.

## Static Feature Detection and Pose Optimization

For each frame of the image, dynamic points and the static points from background image are distinguished. Because of the change of the three-dimensional position of the dynamic feature point, the projection point should be far away from the corresponding feature point when dynamic feature point of the ***(n-1)***th frame is projected onto the ***n***th frame picture. For the static feature point, the re-projection error should be small. Based on this principle, an algorithm is designed to distinguish dynamic feature points from static feature points.

### Initialization

The first step of the proposed algorithm is the initialization process. The image feature of each frame is extracted by using the ORB and SURF feature extraction algorithm, respectively, under a variety of complex environment. For the both methods, the feature points extracted using ORB are less than that of SURF but the speed is faster. The two extraction methods can be selected according to application background and real-time requirements. The extracted feature points are used as the data source for the whole system. These data can be analyzed and the dynamic feature points and the static feature points can be distinguished.

**Figure 2:**
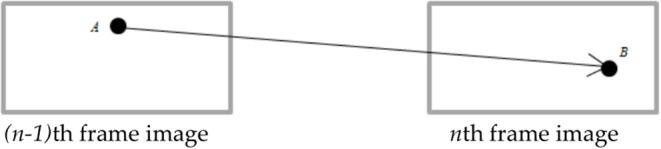
The corresponding feature.

Firstly, after extracting the image feature information of each frame, the data association of the adjacent frame is realized by using the existing matching algorithm, that is, the corresponding feature points between the two frames are found. For example, there is the feature point ***A*** in the ***(n-1)***th frame, then the position of ***A*** will be at the position of the feature point ***B*** in the nth frame after one frame changes, which is shown in Figure 2. Although there exits the mismatching result, the matching result is not processed here. The result is directly passed to the homography matrix. According to ***P***_**2**_ **=** ***HP***_**1**_, homography matrix between two frames is obtained easily by using RANSAC.

**Figure 3:**
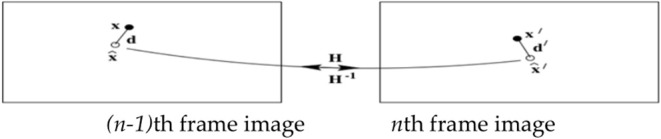
The re-projection error.

Then the static feature points and the dynamic feature points obtained by Brute force method will be separated by using the re-projection error method. The re-projection error is shown in the Figure 3. Its expression is as follows.

(1) $$\begin{array}{l} d{\left(x,\hat{x}\right)}^{2}+d{\left(x^{\prime },{\hat{x}}^{\prime }\right)}^{2} \end{array}$$

According to the re-projection error, the feature points extracted from the ***(n-1)***th frame are projected onto the ***n***th frame image, and the distance between the corresponding point and the projection point of the ***(n-1)***th frame image at the ***n***th frame image is compared. The feature point obey the Gaussian distribution. The Mahalanobis distance between the projection point and the corresponding point is less than a certain value. If the feature point is greater than the Mahalanobis distance, this feature point may be a dynamic point or a mismatch point, which is called an uncertain point. If the feature point is less than the Mahalanobis distance, this feature point may be a static point. It will be regarded as an initial value of a static point which is prepared for the next step. So the whole initialization process is completed, and the process is shown in Table 1.

**Table 1:** Initialization process.

**Initialization process (dynamic feature points, static feature points, the relationship between frame and frame)**
Feature point extraction: keypoints_1,keypoint_2
Feature descriptor: descriptors_1,descriptors_2
Feature matching by Brute Force method: good_matches
The Homography matrix is obtained by giving the matching result to the Homography matrix frame: Initial_***H***
Initial_H(that is, the relationship between the frame and the frame is initialized)To find the projection point by ***H***
Compare the projection point with the corresponding point of Maha distance: diff
Define an error threshold: ***θ***
**For** *i* = 1: *diff.rows*
**If** (*diff* < ***θ***)
The feature point is a static point
—>initial_S_point
**Else**
The feature point is the dynamic point
—>Initial_D_point
**End for**

### Texture Constraint

In adjacent frames, the static point and its re-projection point change very little, and the texture difference is very small, while the dynamic point and its re-projection point change greatly, and the texture difference is large.

The interframe difference method is used to register the background of several successive images, compensate the difference of moving background. The problem of target detection is transformed into the problem of moving target detection in static background, and the moving object is separated by the difference images of adjacent frames. Let the binary image ***I***_***k*−1**_***(x,y)*** and ***I***_***k***_***(x,y)*** be the registered image of the two adjacent frames, and the binary image of the moving object can be obtained by processing the interframe difference and threshold.

(2) $$\begin{array}{l} d_{k-1,k}\left(x,y\right)=\{\begin{array}{l} 1,\left|I_{k}\left(x,y\right)-I_{k-1}\left(x,y\right)\right|>T\\ 0,other\text{wise} \end{array} \end{array}$$

Theoretically in the difference binary image ***d***_***k*−1, *k***_***(x,y)***, only the pixel position in the area covered by the moving object is not zero. However, in practice, because there exist high frequency noise, illumination change and the subtle change of the background in the image and registration will also cause errors at the same time, many pixels are non-zero in the binary image besides moving objects, that is, there exist noisy points. And the method can only segment the contours of the moving objects.

In this paper, frame difference method is used to complete the texture constraint of feature points. The collected picture is RGB three-channel map, the texture constraint will be completed by using three-channel (RGB). The two adjacent frames are sampled in three channels, and the different weighted processing is used according to the sensitivity of each channel. The weighting ratio is set to be 1 and sum operation is done. Let the threshold value be ***T***. If the value of three-channel doing the difference and weighted summing is greater than this threshold ***T***, it demonstrates that the two points do not correspond to the same point. Otherwise, the two points correspond to the same point. The equation is as follows.

(3) $$\begin{array}{l} d_{n}(x,y)=\alpha (\text{ ​​}|\text{​​ }R_{n}(x,y)-R_{n-1}(x,y)\text{ ​​}|\text{​​ })+\beta (\text{ ​​}|\text{​​ }G_{n}(x,y)\\ \;-G_{n-1}(x,y)\text{ ​​}|\text{​​ })\text{+}\gamma (\text{ ​​}|\text{​​ }B_{n}(x,y)-B_{n-1}(x,y)\text{ ​​}|\text{​​ }) \end{array}$$

(4) $$\begin{array}{lll} \alpha +\beta +\gamma & = & 1 \end{array}$$

If ***d*** < ***T***, it means that the two points correspond to the same spatial point, and vice versa, it means that the two points correspond not to the same spatial point. Where ***d***_***n***_ is the texture error of two frames, **α,β,γ** are the weight parameters.

The original frame difference method is no longer used to traverse the whole image pixel, but the feature point. Compared with million pixels of each frame, the feature points are only a few thousand or hundred of feature points, the amount of the calculation is greatly reduced. So the designed texture constraints can be integrated into the feature point classification system proposed in this paper.

### Area Constraint

**Figure 4:**
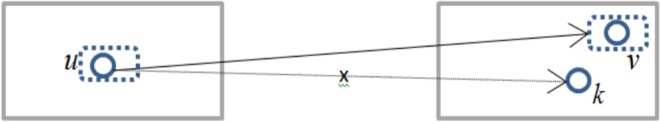
Area constraint diagram.

In order to reduce the mismatch, a region constraint method is designed based on the principle of the constraint line in this paper (Zou and Tan, 2013). Block constraint is mainly used. Assume that there are two candidate points on the nth frame image corresponding to the ***(n-1)***th frame image. In this paper, a constraint window is set, which is shown in Figure 4.

When ***v*** and ***u*** are in the same window, ***v*** and ***u*** are matched each other. When ***k*** is beyond this window, ***k*** and ***u*** do not match. In order to adapt a variety of environment, the size of this active window is changed. It not only improves the accuracy of matching, but also ensures the matching points not to be too sparse. The difference square of the pixel coordinates ***u*** and ***v*** between each matching point of the adjacent image frame are solved when a window is created. Because the difference between the matching point may be negative, the square operation is added. The maximum square of difference value between the adjacent frame ***u*** and ***v*** is obtained by traversing the image frame, respectively. The active window with region constraint is built by obtaining the maximum value, and the length and width of the window are determined by the maximum value of ***u*** and ***v***, respectively. The expression is as follows:

(5) $$\begin{array}{l} f\left(P_{n},P_{n-1}\right)=\{\begin{array}{cc} \text{Correct correspondence} & \text{ ​​}|\text{​​ }u_{n}-u_{n-1}\text{ ​​}|\text{​​ }<\theta U_{\max}\text{ and }v_{n}-v_{n-1}\text{ ​​}|\text{​​ }<\eta V_{\max}\\ \text{Error correspondence} & other\text{wise} \end{array} \end{array}$$

Where ***P***_***n***_***(u***_***n***_***,v***_***n***_***)*** and ***P***_***n*−1**_***(u***_***n*−1**_***,v***_***n*−1**_***)*** are the corresponding points between the adjacent frames, ***U***_***max***_ and ***V***_***max***_ are the square of the maximum difference, **θ** and **η** are the proportional coefficient. In Equation (5), a window with length ***U***_***max***_ and width ***V***_***max***_ is established which realizes the region constraint for the matching point. Here, the size of the active window is automatically adjusted according to corresponding points between frame and frame influenced by the noise, while the proportional coefficient is set in order to ensure the sparseness between frame and frame. So the whole system automatically adjusts the size of the window in different environment, which can ensure the adaptability of the system.

### Static Feature Detection

According to the initialization process in section Initialization, the initial homography matrix ***H***, the initial static points and the uncertain points can be obtained. In this section, the initial static feature points are filtered by using the proposed method. The spatial position represented by the static point does not change, so the interference is very small in the short frame time. The change of three color channels is also very small, so the judgment is more accurate. The overall process of the static target is shown in Table 2.

**Table 2:** The steps of the static target processed.

**The separation process of static feature points**
Initialize static feature points: Initial_S_point
Static Constraints:
Set up ***θ**, **η***
Traverse the image ***U***_***max,***_ ***V***_***max***_
**For** *i* = 1: *matches.size()*
If the difference square of the adjacent coordinate points
***XX**^2^*<***θU**_***max***_**,YY**^**2**^* **< ηV**_***max***_
Two points correspond
Generate a new match pair: matches1
**Else**
If two points are mismatched, eliminated
**End if**
**End for**
Texture Constraints:
Set up ***α,β,γ***
Traverse the image to get the texture error ***d**_*n*_*
**For** *i* = 1: *matches.size()*
**If** ***d**_*n*_* **<** ***T***
Two points correspond
Generate a new match pair matches2, get the final static feature point
Static_point
**Else**
If two points are mismatched, eliminated
**End if**
**End for**
The final static match point is given to the homography matrix
Perform RANSAC algorithm
Get the conversion relationship between frame and frame Homography

First, the initialized static point is used as input. Because the attributes of static features do not change much, the static region constraints are combined with the region constraints module in section Initialization. And the region constraint window is relatively small, which is more accurate for mismatches. However, there are still some mismatches, and the screening results can not meet the actual requirements. Therefore, this paper introduces the method of texture constraints.

The three color channels can greatly reduce the error caused by illumination because of the change of pose. Secondly, the whole frame is processed by a frame and a frame. The pose of the static target is changed a little in the view angle. So the texture constraint is added, which can greatly reduce the mismatching result. According to the two screening steps proposed in this paper, a set of accurate static feature points can be obtained. Then the extraction of static feature points is completed. The extracted static points can be pass to the homography matrix again and the homography matrix is solved by the RANSAC algorithm. In this case, the homographic matrix ***H*** can accurately express the transformational relation between the two frames, and the homographic matrix can be decomposed to obtain the transformation matrix ***R*** and translation matrix ***T*** between the frame and frame. It can achieve the function of visual odometer. But in this experiment, only the change of the relationship between the two frames is needed, so the homographic matrix ***H*** doesn't need to be decomposed. The homography matrix obtained again will be used in the dynamic target separation.

## Constraint

### Double Texture Constraint

**Figure 5:**
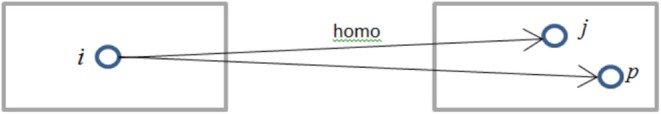
Double texture.

The texture problem of dynamic feature points is different from that of static feature detection. Each dynamic point has two points, one is the projection point, the other is the matching point. In Figure 5, ***i*** is a dynamic feature point, then ***j*** is the point which ***i*** is projected by the homography matrix in section Static Feature Detection, and ***p*** is obtained by descriptor matching of the adjacent two frames.

In the two frame images, ***i*** and ***j*** correspond to the same spatial position. The dynamic point ***i*** is moved from the projection position ***j*** to ***p***. The difference between ***i*** point of the ***(n-1)***th frame and the ***p*** point of the ***n***th frame is small, while the difference of the texture between the ***i*** and ***j*** points is very big (although sometimes the background is similar to the texture of the moving object). According to this principle, a double texture constraint is designed to separate dynamic feature points by the texture constraint in section Area Constraint.

According to section Texture Constraint, the first texture constraint is the same as the static texture constraint, and the texture check between matching points is done. In the second texture constraint, the texture constraint between the re-projection points is compared. The expression is as follows.

(6) $$\begin{array}{l} dd_{\text{n}}=\alpha (\text{ ​​}|\text{​​ }i(x,y)-j(x,y)\text{ ​​}|\text{​​ })+\beta (\text{ ​​}|\text{​​ }i(x,y)\\ \;-j(x,y)\text{ ​​}|\text{​​ })\text{+}\gamma (\text{ ​​}|\text{​​ }i(x,y)-j(x,y)\text{ ​​}|\text{​​ }) \end{array}$$

(7) $$f\left(x,y\right)=\{\begin{array}{cc} \text{Dynamic point} & d_{n}<T\text{ and }dd_{n}>T\\ \text{Error correspondence} & other\text{wise} \end{array}$$

Where ***dd***_**n**_ is the dynamic texture error of two frames, ***f(x,y)*** completes the judgment of the nature of the point ***(x,y)***.

In **Figure 7**, ***j(x,y)*** is the projection point of the previous frame image ***i(x,y)***, where the coordinates ***j*** can be calculated from the obtained homography matrix. Other parameters can be given by static texture constraint.

### Dynamic Detection

This section will introduce the separation of the dynamic feature points. The initialization of the dynamic feature points can be obtained by the initialization process in section Initialization, and the homographic matrix ***H*** between frame and frame can be obtained by separating the static feature points. The two data sets are regarded as the input values of the dynamic separation. The initial dynamic points contain the dynamic points and the mismatch points. In this paper, the mismatch points will be filtered by the proposed method in section Double Texture Constraint and the accurate dynamic points are separated. Firstly, uncertain points can be filtered out by the second re-projection error method proposed in this paper. The accurate dynamic feature points can be obtained because the filtering is performed by an accurate homographic ***H***. But the accuracy is not good enough to cause the converge. So a dynamic region constraint method similar to the static region constraint is introduced. But the dynamic feature points in the actual space will change greatly, so the ratio parameters of regional window will be changed greatly. Otherwise the real dynamic feature points will be regarded as mismatch points and removed, and the separation of dynamic points can not be completed. The size of the window is decided by the distance of all dynamic feature points, which can ensure a certain number of dynamic points. Some of the dynamic points that does not meet the region are removed by the constraints of dynamic region, but some of the error points are still retained. According to the dual texture constraint method in section Double Texture Constraint, the three color channels are performed weighted constraint at the same time. Uncertain points and their own projection points are compared with the texture of the corresponding point. This method can realize the interaction of features and texture, and achieve the separation of dynamic feature points. The process is shown in Table 3.

**Table 3:** The separation process of the dynamic target.

**The initialization process of the feature points**
Initial value: ID_point Homography
Second re-projection error
Double texture constraint
Set up ***α,β,γ***
Traverse the image to get the texture error ***d**_*n*_,**dd**_*n*_*
**For** *i* = 1: *matches.size()*
**If** ***d**_*n*_* < ***T*** and ***dd**_*n*_*>***T***
Get the final static feature point Dynamic_point
**Else**
If two points are mismatched, eliminated
**End if**
**End for**

Both the separation of the static points in section Static Feature Detection and Pose Optimization and the separation of the dynamic points in section Experiment Results use the region constraint and texture constraint. Although the two separation is similar in principle, the implementation method is different. The two constraint methods in the static region play an important role, while the filtering effect of the region constraint in the dynamic separation is not obvious, which is mainly caused by the attribution of the dynamic points. Because the feature matching coordinate is computed by the two constraint methods and the descriptor is not involved, so the computation time is not long. The experiment will be performed in the next section.

## Experiment Results

**Figure 6:**
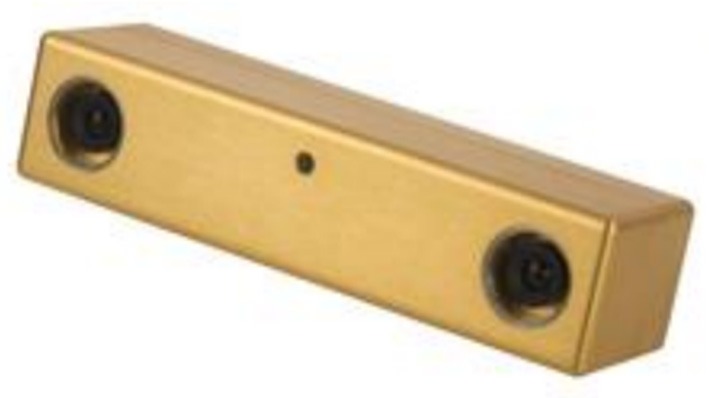
Bumblebee binocular camera.

The computer operated in the experiment is Windows 64-bit, operating system AMD A6-6400 APU with Radeon (tm) HD Graphics (3.90 GHz), 4GB RAM. The visual sensor in this paper is used Bumblebee binocular camera shown in Figure 6. Please note that Bumblebee has two cameras, but only one of them is used in the experiment.

The frame sequence of the environment is obtained off-line. The resolution of the frame image is 1,024 × 768, 15 frames/s. The obtained image frame is directly used in the experiment. Four experiment scene is designed to test the effect of the extracted feature by using the proposed method based on ORB and SURF. The blue dot in the experiment represents the static points obtained by the experiment and the red dot represents the dynamic points, which are shown in the video.

Experiment 1: An object with a large area moves in a static background. In the experiment, the camera keeps stationary, that is, the environment background does not change, and the person moves as a dynamic target at a relatively close distance of the camera, the close distance is within 1 m. In this scene, the classification effect of the environment feature is tested by the proposed method in this paper when the object with a large area (the object with a large area is the person in this experiment) moves close to the camera.

**Figure 7:**
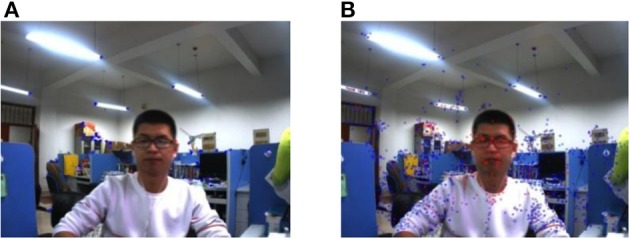
An object with a large area moves close to the camera in the static background. **(A)** The proposed method based on ORB. **(B)** The proposed method based on SURF.

In **Figure 9**, the image on the left is the experiment result of the classification by using the proposed method based on ORB, and the image on the right is the experiment result of the classification by using the proposed method based on SURF. At the same time, the average consuming time of each step is summarized, which is shown in Table 4. The extraction of the environment feature is completed by using the second re-projection error integrated with the texture detection based on ORB algorithm and SURF algorithm. The experiment results show that the two proposed methods in this paper can complete the Feature classification of the environment feature. Table 4 shows the speed of the feature classification by using the second re-projection error integrated with the texture detection method based on ORB is much faster than that of the proposed method based on SURF. However, Figure 7 shows the classification error by using the proposed method based on ORB is smaller than that of the proposed method based on SURF.

**Table 4:** The experiment 1 results using the proposed method based on ORB and SURF.

**Time (ms/frame)**	**Feature point extraction**	**Feature point matching**	**Feature classification**
ORB	527.17	26.57	10.96
SURF	8378.05	422.55	24.88

Experiment 2: Multiple objects in the far distance move in a static background, the far distance is about 5–8 m. The longest distance in the experiment is about 8 meters, which can ensure the experiment effect. In the experiment, the camera remained stationary, so the environment background also remains stationary. Four students in the far distance walk through the camera in turn, while the camera collects the dynamic scene with far distance from one dynamic object to multiple dynamic objects. When the crowd has displacement in the plane direction of the camera, the effect of this method is acceptable, but when the direction of crowd movement is perpendicular to the camera, the moving person may be misjudged as a static point.

**Figure 8:**
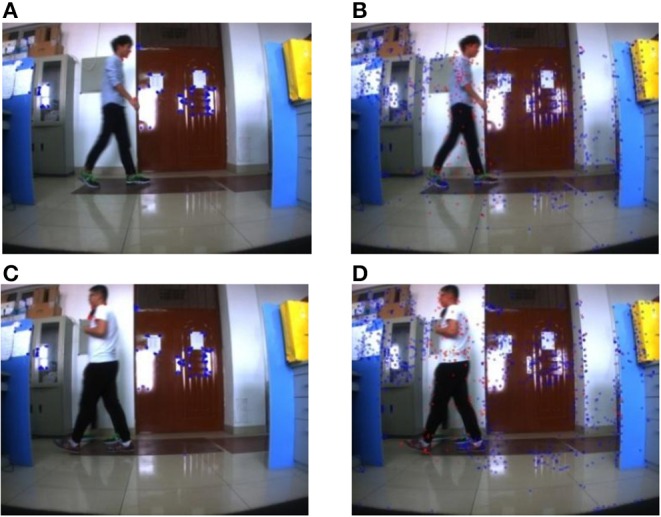
Multiple moving objects with far distance in a static background (1). **(A)** ORB method. **(B)** SURF method (One student). **(C)** ORB method. **(D)** SURF method (One student).

**Figure 9:**
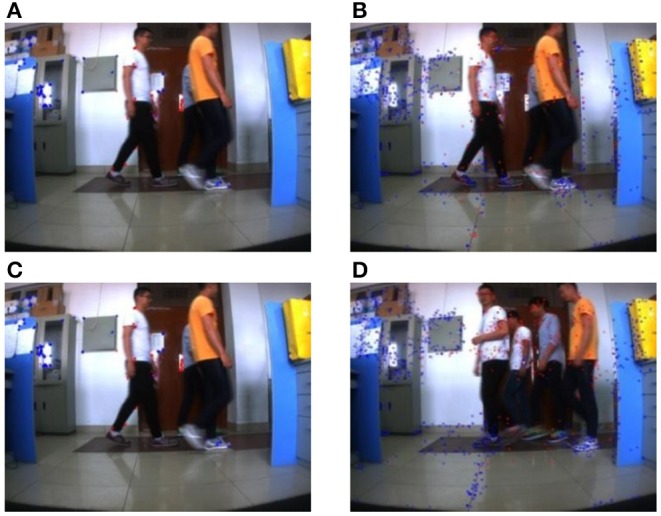
Multiple moving objects with far distance in a static background (2). **(A)** ORB method. **(B)** SURF method (Three student). **(C)** ORB method. **(D)** SURF method (Four student).

Figures 8A,C, 9A,C are the experiment results in the far distance from one dynamic object to multiple dynamic objects by using the proposed method based on ORB. Figures 8B,D, 9B,D are the experiment results in the far distance from one dynamic object to multiple dynamic objects by using the proposed method based on SURF. At the same time, the average consuming time of each step is summarized, which is shown in Table 5. The experiment results demonstrate that the proposed method in this paper can realize the feature classification whether or not there exist one or multiple moving objects with far distance in a static background. And Table 5 shows the speed of each step for Experiment 2.

**Table 5:** The experiment 2 results using the proposed method based on ORB and SURF.

**Time (ms/frame)**	**Feature point extraction**	**Feature point matching**	**Feature classification**
ORB	794.78	26.46	9.86
SURF	8229.56	362.00	23.24

Experiment 3: An object with a large area moves in a dynamic background. The pose of the camera changes when the camera extracts the frame image. The camera and the moving object can move without the limitation of position and speed.

**Figure 10:**
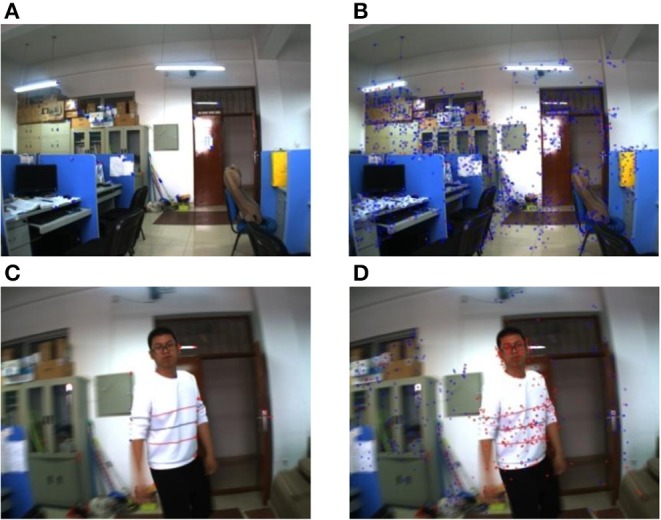
One object moves close to the camera in the dynamic background. **(A)** ORB method. **(B)** SURF method. **(C)** ORB method. **(D)** SURF method.

The experimental results are shown in Figure 9. Figures 10A,C are the experiment results of the classification by using the proposed method based on ORB, and Figures 10B,D are the experiment result of the classification by using the proposed method based on SURF. The Figures 10A,B are the results of the initial state of the environment by using the proposed method based on ORB and SURF in this paper. At the same time, the average consuming time of each step is summarized, which is shown in Table 6. The experimental results demonstrate that both of the methods can identify the static point. Figures 10C,D is the experiment results of one dynamic object by using the proposed method based on ORB and SURF in the paper. Both of the two proposed methods based on ORB and SURF can successfully identify the static and dynamic feature points. Figure 10 shows the classification feature by using the proposed method based on SURF is more than that of the proposed method based on ORB. And Table 6 shows the speed of the feature classification using the two proposed methods based on ORB and SURF.

**Table 6:** The experiment 3 results using the proposed method based on ORB and SURF.

**Time (ms/frame)**	**Feature point extraction**	**Feature point matching**	**Feature classification**
ORB	627.58	24.47	84.03
SURF	7767.62	265.42	106.13

Experiment 4: Multiple objects in the far distance move in a dynamic background. The pose of the camera changes when the camera extracts the frame image. The camera and all the moving objects can also move without the limitation of position and speed.

**Figure 11:**
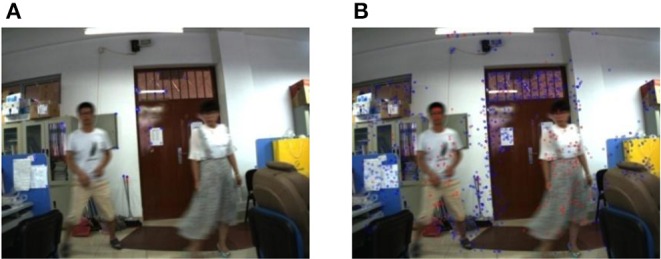
Multiple moving objects with far distance in a dynamic background. **(A)** ORB method. **(B)** SURF method.

The experimental results are shown in Figure 11. Figure 11A is the experiment result of the classification by using the proposed method based on ORB, and Figure 11B is the experiment result of the classification by using the proposed method based on SURF. At the same time, the average consuming time of each step is summarized, which is shown in Table 7. Figure 11 shows the experiment results show that both of the two proposed methods can complete the feature classification of the environment. However, Table 7 shows the speed of the classification by using the second re-projection error integrated with the texture detection method based on ORB is much faster than that of the proposed method based on SURF.

**Table 7:** The experiment 4 results using the proposed method based on ORB and SURF.

**Time (ms/frame)**	**Feature point extraction**	**Feature point matching**	**Feature classification**
ORB	645.76	24.60	91.87
SURF	10052.98	512.76	131.22

The experiments show that the feature points can be classified accurately for one object or multiple objects whether or not in the static environment or in the dynamic environment.

**Figure 12:**
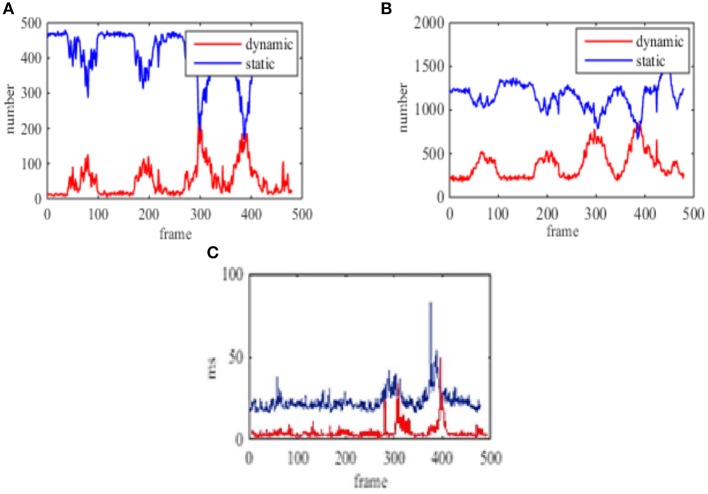
The number of feature points of multiple moving objects in the far distance and the time of each frame in the static background. **(A)** The classification number of the static and dynamic points based on ORB. **(B)** The classification number of the static and dynamic points based on SURF. **(C)** The processing time based on ORB and SURF, Red curve is ORB, blue curve is SURF.

**Figure 13:**
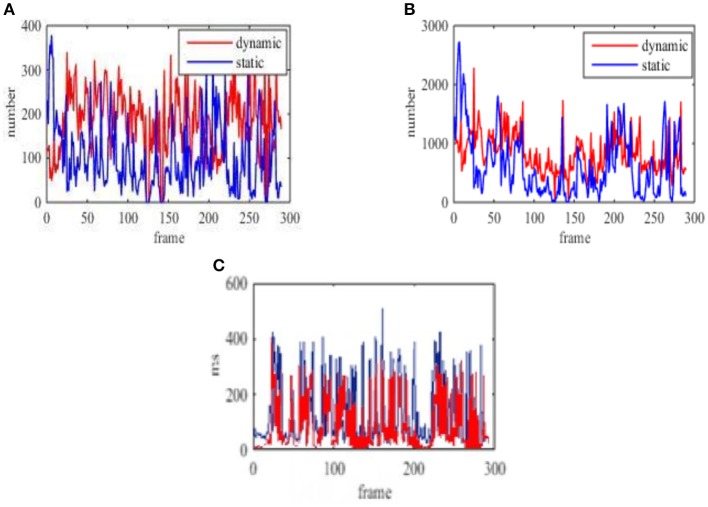
The number of feature points of multiple moving objects in the far distance and the time of each frame in the dynamic background. **(A)** The classification number of the static and dynamic points based on ORB. **(B)** The classification number of the static and dynamic points based on SURF. **(C)** The processing time based on ORB and SURF, red curve is ORB, blue curve is SURF.

In addition, the number of static points and dynamic points are counted based on the classification of each frame image in experiment 2 (shown in Figure 12) and experiment 4 (shown in Figure 13). The consumed time for each frame classification algorithm (excluding feature extraction and matching time) is also compared. Here the time of the feature points processed is considered because the classification algorithm is proposed in this paper. Feature extraction and matching are the application of existing methods. At the same time, the average consuming time of each step in experiment 4 is summarized, which is shown from Table 4 to Table 7. Then the real-time performance of the four environment by using the proposed method in the paper can be compared.

**Figure 14:**
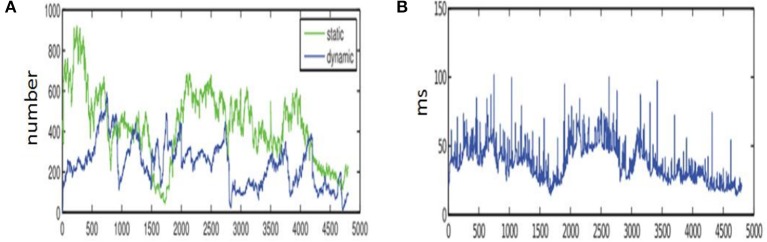
The experiment results of Zou and Tan (2013). **(A)** The classification number of the static and dynamic points in Zou and Tan (2013). **(B)** The running time of the static and dynamic points in Zou and Tan (2013).

In Zou and Tan (2013), the KLT tracker is used to complete the feature extraction by using multiple visual sensors. And the feature classification is realized by using the re-projection error under the GPU acceleration, which is more suitable for the feature detection of the high-speed dynamic environment. The results in Zou and Tan (2013) is shown in Figure 14. This paper is based on the study of Zou and Tan (2013). Different from Zou and Tan (2013), this paper only uses a sensor to achieve the feature classification, which is more practical and convenient for the SLAM. Figures 12, 14A shows the number of classification is much more than that of Zou and Tan (2013) when SURF is used to extract the feature of the environment. Although, the number of feature classification using ORB to extract the feature of the environment is less than that of using SURF, the running time using ORB is only a dozen milliseconds. Tables 4–7 shows that the time of processing feature extraction using SURF is 130 ms average per-frame. But the time of processing feature extraction using ORB is 91.87 ms average per-frame. At the same time, Figures 12, 14B also shows it conforms to the require of real-time just as Zou and Tan (2013). The proposed algorithm in this paper can adapt to the dynamic environment with all kinds of speed, and has good effect for the moving objects with large area. So the second re-projection error method integrated with the texture detection proposed in the paper can obtain good performance for the feature classification of the dynamic environment by using a vision sensor.

Figures 12A,C show the number of feature points and processing time for each frame using the proposed method based on ORB in the static background. Figures 12B,C show the number of feature points and processing time for each frame using the proposed method based on SURF in the static background.

Figures 13A,C show the number of feature points and processing time for each frame using the proposed method based on ORB in the dynamic background. Figures 13B,C show the number of feature points and processing time for each frame using the proposed method based on SURF in the dynamic background.

## Conclusion and Future Work

This paper presented a new classification algorithm of feature points in dynamic environment by using a visual sensor. The designed three framework is initialization, static targets detection and dynamic targets detection, which can complete the classification of dynamic feature points and static feature points in various complex environments. And the transformation relationship between frame and frame is optimized. The experiment results demonstrate that the feature points can be classified accurately for one object or multiple objects whether or not in the static environment or in the dynamic environment. And the proposed algorithm in this paper has good effect for the moving objects with large area. So the second re-projection error method integrated with the texture detection proposed in the paper can obtain good performance for the feature classification of the dynamic environment by using a vision sensor. The experiment was carried out indoors, and the method can work normally under sufficient sunshine or light. In order to improve the illumination adaptability of this method, RGB three-channel can be optimized to RGB-HSV six-channel in subsequent experiments, and the weight of H-channel can be increased to improve the illumination adaptability.

In future work, we will further study the image processing and screening, and apply the proposed algorithm to the SLAM.

## Data Availability Statement

The datasets for this article are not publicly available because: [The dataset contains some private information]. Requests to access the datasets should be directed to SWe, swen@ysu.edu.cn.

## Ethics Statement

Written informed consent was obtained from the individual(s) for the publication of any potentially identifiable images or data included in this article.

## Author Contributions

SWe and ZZ put forward this idea and made a rigorous theoretical analysis. Under the guidance of SWe, ZZ completed the experiment and collected data. SWa, XZ, and DZ compiled and analyzed the experimental results and data to form the final manuscript. All authors provided critical feedback and made improvements and optimizations.

### Conflict of Interest

The authors declare that the research was conducted in the absence of any commercial or financial relationships that could be construed as a potential conflict of interest.
